# A Mach-Zehnder Fabry-Perot hybrid fiber-optic interferometer operating at the thermal noise limit

**DOI:** 10.1038/s41598-022-16474-y

**Published:** 2022-07-15

**Authors:** Nabil Md Rakinul Hoque, Lingze Duan

**Affiliations:** Department of Physics and Astronomy, The University of Alabama in Huntsville, 301 Sparkman Drive, Huntsville, AL 35899 USA

**Keywords:** Optical sensors, Imaging and sensing, Optical metrology, Techniques and instrumentation

## Abstract

A new type of interferometric fiber sensor based on a Mach-Zehnder Fabry-Perot hybrid scheme has been experimentally demonstrated. The interferometer combines the benefits of both a double-path configuration and an optical resonator, leading to record-high strain and phase resolutions limited only by the intrinsic thermal noise in optical fibers across a broad frequency range. Using only off-the-shelf components, the sensor is able to achieve noise-limited strain resolutions of 40 f$\varepsilon$/$\sqrt{(}Hz)$ at 10 Hz and 1 f$\varepsilon$/$\sqrt{(}Hz)$ at 100 kHz. With a proper scale-up, atto-strain resolutions are believed to be within reach in the ultrasonic frequency range with such interferometers.

## Introduction

Fiber-optic interferometers have garnered tremendous interest in recent years due to their potential applications in optical sensing^1^, fiber-optic communications^2^, optical computing^3^, and biomedical imaging^4,5^. Passive interferometric fiber sensors (IFS), in particular, are capable of reaching extremely high signal resolutions, making them especially suitable for developing ultra-sensitive optical sensors^6–9^. Fundamentally, all IFS are built upon the same operating principle, i.e., probing optical phase/frequency fluctuations induced by external measurands (e.g., strain, temperature, pressure, etc.) through optical interference^1^. In order to optimize the ability of an IFS to resolve small signals, one needs to *i)* maximize the response of the sensor to external perturbations (i.e., sensitivity) and *ii)* minimize unwanted noise.

The first goal can be accomplished by using interferometric schemes featuring sharp phase/frequency discrimination. Over the years, several ultra-sensitive IFS techniques have been demonstrated, including $\pi$-phase shifted fiber Bragg gratings ($\pi$-FBG)^10–13^, slow-light FBG^14–17^, and long fiber Fabry-Perot interferometers^18–23^. Meanwhile, considerable effort has also been dedicated to lowering the noise. Since the noise of the interrogation laser typically dominates in a passive-IFS scheme, most of the recent research has been focused on either developing novel low-noise lasers^24^ or improving the laser-stabilization techniques^25^.

Ultimately, however, the resolution of IFS is limited by the intrinsic thermal noise of optical fibers. There are two types of thermal noises in fibers. The *thermodynamic* noise (also known as the thermoconductive noise), which features a quick roll-off at high frequencies, typically dominates at frequencies above 100 Hz^26,27^. The *thermomechanical* noise, which is of a 1/*f* spectral characteristic, is the predominant mechanism at low frequencies (e.g., < 10 Hz)^28,29^.

Achieving thermal noise-limited fiber-optic sensing is both attractive and challenging: attractive because it represents the maximum resolving power a sensor can possibly achieve; challenging because reaching the minuscule thermal noise requires a sensing system to have *both* an extremely high sensitivity and a very low system noise^30–32^. Over the last three decades, there has been a continued effort to develop fiber-optic sensors that can operate at the thermal noise level^1,33–38^. Generally, two distinctive approaches have been taken to accomplish this goal: *i)* frequency discrimination and *ii)* phase discrimination. In a frequency-discrimination scheme, an optic resonator such as a fiber Bragg grating (FBG)^37^ or a fiber Fabry-Perot interferometer (FFPI)^38^ is employed to create a sharp spectral feature (i.e., a resonance peak) that can be used as a highly sensitive optical-frequency discriminator. The advantage of this approach is that the sensor itself can be very compact, typically on the order of a meter or less. The disadvantage, however, lies in their inability to distinguish the sensing signal from laser noise, which often makes the interrogating laser the greatest liability of the overall sensor resolution^39^. As a result, in order to attain thermal-noise-limited operation with a frequency-discrimination scheme, either an ultralow-noise laser^37^ or a highly sophisticated laser frequency-stabilization system^30,38^ has to be deployed. Meanwhile, a phase-discrimination scheme leverages the phase sensitivity of a traditional double-path interferometer, such as the Michelson^36^, the Mach-Zehnder^35^, or the Sagnac configuration^33^. It has a much lower requirement on the interrogation laser because the phase/frequency noise of the laser is a common-mode noise in these interferometers. On the other hand, phase-discrimination sensors are often quite bulky, with arm lengths well exceeding tens or even hundreds of meters in order for them to attain sufficient phase sensitivity^35,36^. They are not only difficult to package but also highly susceptible to environment-induced fluctuations.

In this paper, we report the demonstration of a new type of ultrahigh-resolution IFS: a Mach-Zehnder Fabry-Perot (MZ-FP) hybrid interferometer. The interferometer combines a traditional double-path configuration with fiber-optic resonators to overcome the shortcomings of the prior IFS schemes. This leads to a compact IFS system capable of operating at the thermal noise level while interrogated by an off-the-shelf commercial diode laser. Moreover, a soil-based isolation system has been devised to help achieve record-level strain resolutions across a broad frequency range.

## Experimental method and setup

**Figure 1:**
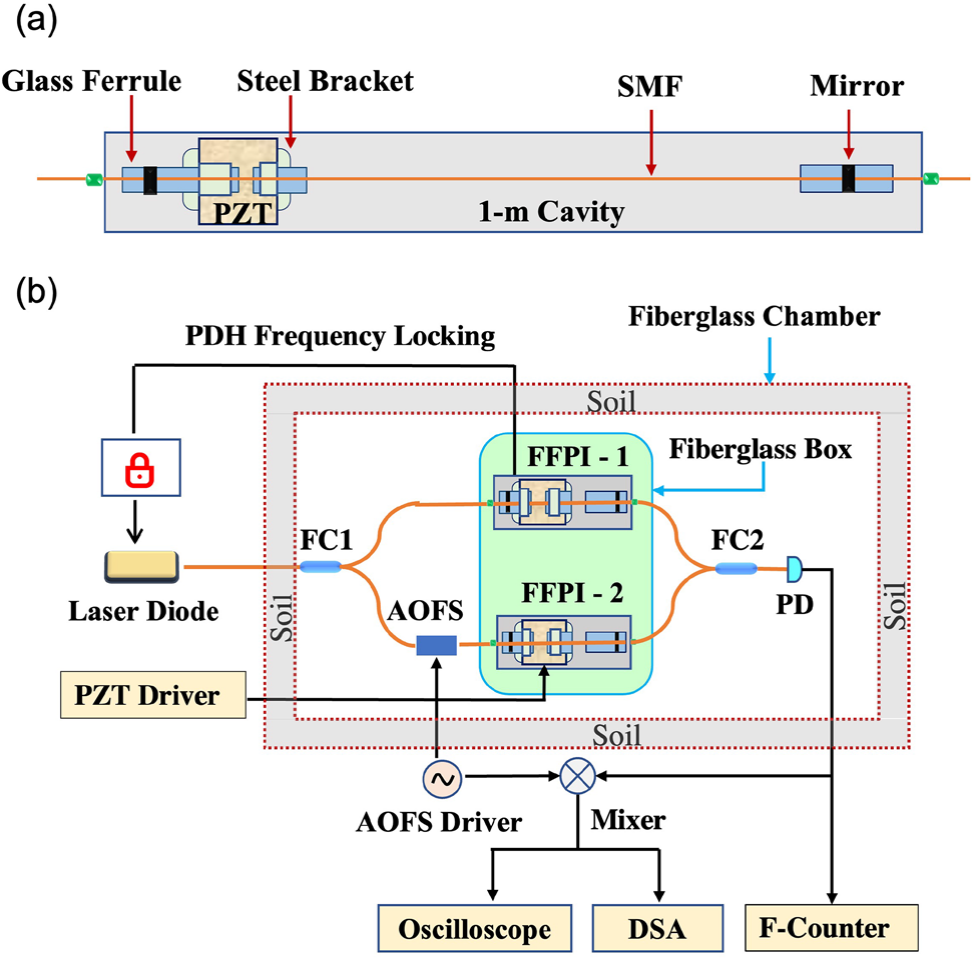
(**a**) A schematic diagram of the FFPIs used in the experiment. (**b**) System layout of the MZ-FP hybrid interferometer.

The idea behind the MZ-FP hybrid interferometer is very simple: A double-path interferometer such as a Mach-Zehnder is ideal for mitigating the impact of the laser noise but requires long arms to achieve desired phase sensitivity. Meanwhile, an optical resonator such as a Fabry-Perot offers high sensitivity in a miniature size as it effectively folds a long optical path inside a small package. Now, if we replace the two long arms of a Mach-Zehnder with two identical Fabry-Perots, the resulted hybrid configuration in principle can retain the benefits of *both* schemes. The concept of MZ-FP hybrid interferometers was first conceived by one of us in a previous report^39^. The current paper focuses on its experimental realization.

Figure 1 shows our experimental setup. Two commercial FFPIs (Micron Optics, FFP-SI), as illustrated in Fig. 1a, are identical in configuration and specifications, each with a cavity length of 1 m, a free spectral range of 105 MHz, and a linewidth of 116 kHz. Multilayer dielectric mirrors are coated on both ends of the FFPIs, allowing them to attain a high finesse of about 902. With a meter-long cavity made of single-mode fiber (SMF), each FFPI accounts for an effective fiber path of about 574 m when operating on resonance^39,40^. A piezoelectric (PZT) actuator is attached on each FFPI to allow fine adjustment of the cavity length. The overall experimental layout is shown in Fig. 1b. The interferometer is interrogated by a commercial single-frequency diode laser (RIO, Orion) operating at 1550.1 nm with a sub-1 kHz linewidth. Two fiber couplers, FC1 and FC2, form the double-path configuration for the MZ interferometer, with the two FFPIs, named here as FFPI-1 and FFPI-2, inserted in its two arms. A photodetector (PD) following FC2 probes the interferometric output. When operating on resonance, the insertion loss of the two FFPIs is about 5 dB. The MZ-FP interferometer has an overall insertion loss (under the quadrature condition) of 15 dB due to additional fiber couplers inserted in the interferometer (not shown in Fig. 1b) for PDH locking and signal monitoring purposes.

To ensure optimum operation of this hybrid interferometer, two technical challenges have to be overcome first: *i)* both FFPIs must be able to stay on resonance with the laser *simultaneously* for extended periods of time (minutes or longer), and *ii)* fluctuations caused by ambient environment must be suppressed to below the level of fiber thermal noise. To address the first challenge, a Pound-Drever-Hall (PDH) system is used to lock the laser frequency to a resonance peak of FFPI-1, as shown in Fig. 1b. Moreover, FFPI-1 and FFPI-2 are sealed together in a fiberglass box to keep them under the same isolated environment. By applying a dc voltage on the PZT actuator in FFPI-2, the resonance frequency of FFPI-2 can be tuned to match the frequency of the laser. This allows the laser to be on resonance with both FFPIs at the same time. Since the two FFPIs are packaged together, they experience similar fluctuations, which helps preserve the resonance condition for as long as several minutes even in the absence of a direct frequency locking between FFPI-2 and the laser. Meanwhile, to suppress environment-induced phase fluctuations, the entire MZ interferometer is mounted in a large fiberglass chamber insulated with a 2-inch layer of garden soil in all directions. Soil is chosen here as the insulation material because of its superior thermal and acoustic isolation properties^41^. The chamber is placed on top of a vibration isolator (Minus K, BM-1) to block low-frequency vibrations from the ground.

The relative phase fluctuation between the two FFPIs is an important measure of the stability of the MZ-FP interferometer. To better characterize it, a fiber-coupled acousto-optic frequency shifter (AOFS) is installed in one of the MZ arms. The AOFS is driven by a 50-MHz harmonic signal, which results in a 50 MHz beat note on PD. Part of the beat note is sent to a frequency counter (SRS SR620) for Allen-Deviation measurement. The remaining beat note is frequency-shifted to the baseband by mixing it with the original driving signal in quadrature for phase noise analysis, which is performed by an oscilloscope (Keysight DSOX3034T) and a Fourier-transform dynamic signal analyzer (DSA) (SRS, SR785).

## Experimental results

**Figure 2:**
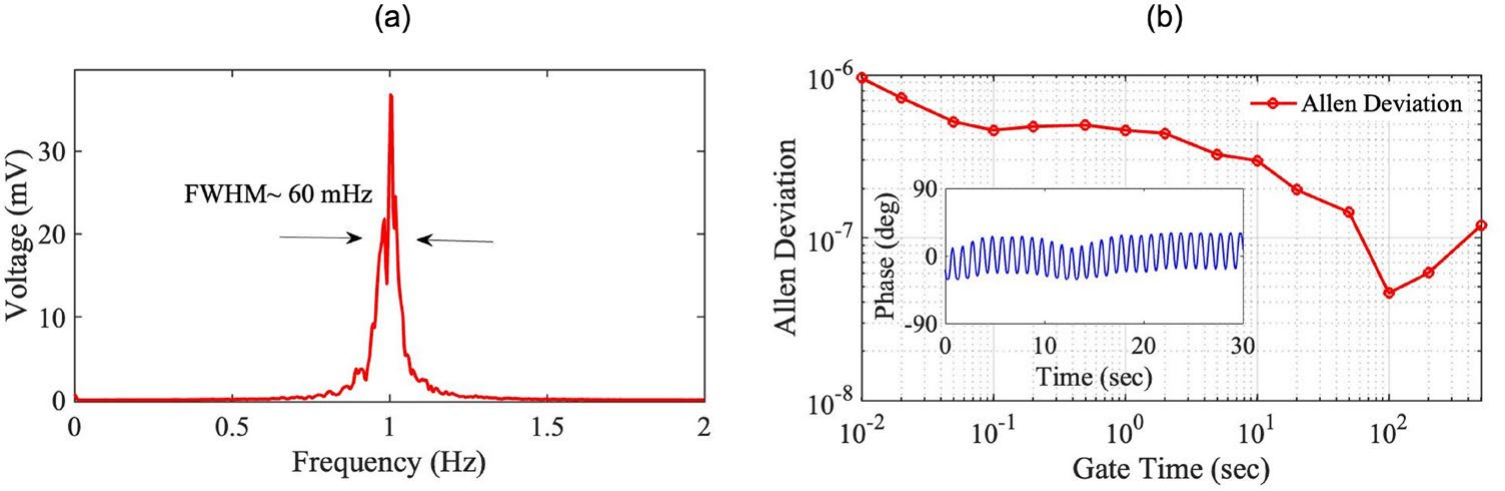
(**a**) A beat note signal between the two MZ-FP arms exhibits a FWHM of about 60-mHz. (**b**) The Allen deviations of the beat note. Inset: Slow fluctuations of the relative phase between the MZ-FP arms near the quadrature point. The 1-Hz phase modulation is intentionally added for calibration purposes.

As pointed out earlier, the proper operation of the MZ-FP hybrid interferometer relies on simultaneous resonance of both FFPIs with the laser. Since the laser is frequency-locked to FFPI-1, the relative phase fluctuations between FFPI-1 and FFPI-2 dictates the effectiveness of the scheme. These fluctuations are imprinted in the 50-MHz beat note as excess frequency noise, which has been carefully measured in various time scales. The results of these measurements are summarized in Fig. 2. Figure 2a shows the spectrum of the beat note, which has a full-width-half-maximum (FWHM) value of 60 mHz. The spectrum is measured by beating the 50-MHz signal down to 1 Hz and then analyzing it with the DSA. The gate time of the DSA is 256 s, leading to a frequency resolution of 3.9 mHz. Time-domain characterization of the beat note has also been carried out using the frequency counter, and the result is shown as Allan deviation in Fig. 2b. Notably here, the Allen deviation reaches its minimum value of $4.56\times 10^-{^8}$ at a gate time of 100 s before it bounces back at longer gate times, indicating the dominance of slow frequency drift. Such a slow drift can be seen in the time domain by monitoring the fluctuation of the baseband beat note under the quadrature condition. A sample of such a measurement is shown in Fig. 2b inset for a duration of 30 s. Here, a 1-Hz phase modulation is intentionally added to the 50-MHz local oscillator with a peak-to-peak amplitude of 50-degree. This generates a 1-Hz oscillation in the beat note, allowing us to calibrate the slow beat-note drift in terms of phase. Overall, we have found that, once optimized, the quadrature condition can typically maintain for several minutes, validating the feasibility of the MZ-FP hybrid scheme. Such a time scale is also evident from the fact that Allan deviations at gate times as long as 500 s have been successfully recorded.

**Figure 3:**
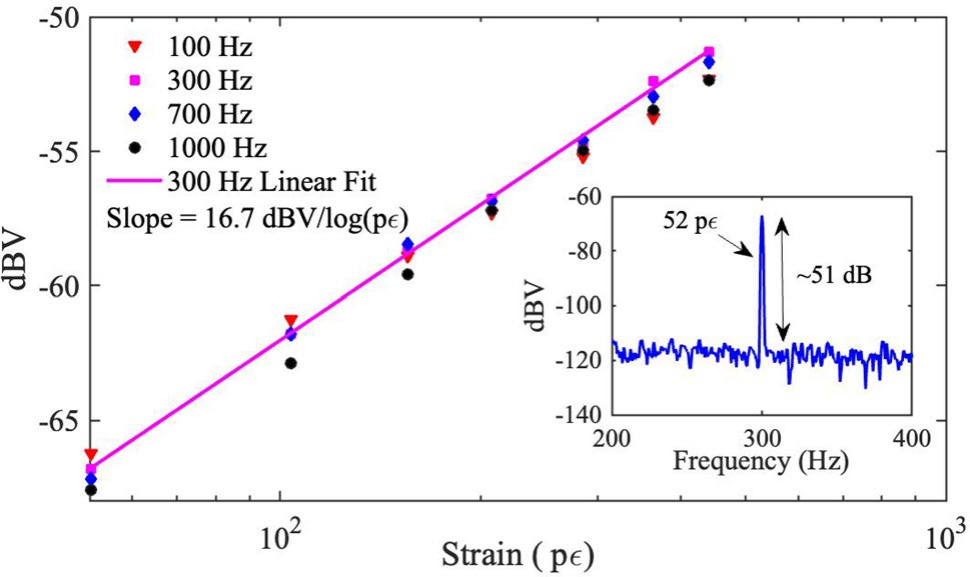
Measured MZ-FP responses under harmonic strain modulations of various amplitudes at 100 Hz, 300 Hz, 700 Hz and 1 kHz. Linear fit and slope are given for data at 300 Hz. Inset: The interferometer output due to a 52-$p\varepsilon$ signal displays a signal-to-noise ratio of $\sim 51$ dB.

To demonstrate the MZ-FP interferometer as a fiber-optic sensor, dynamic strain signals are introduced to one of the FFPIs by applying a harmonic modulation on its PZT actuator, and the resultant interferometric response is monitored. The actual amounts of strain applied on the FFPI are calibrated using the manufacturer-specified PZT response, which is independently verified in our experiment. Figure 3 shows the measured MZ-FP response when this strain modulation is at 300 Hz with various amplitudes. The MZ-FP output displays a good linearity versus the input strain signal on a dB-log scale, as shown by the linear fit and the resulted slope. Figure 3 inset shows the actual strain modulation peak detected at the interferometer output due to a tiny strain amplitude of 52 p$\varepsilon$. This is the lowest strain amplitude we can reliably produce with the PZT actuator and the peak is still 51 dB above the noise floor, indicating a very high level of strain resolution. Similar strain-response measurements have also been taken at other modulation frequencies, e.g., 100 Hz, 700 Hz and 1000 Hz, and the corresponding data points are included in Fig. 3.

**Figure 4:**
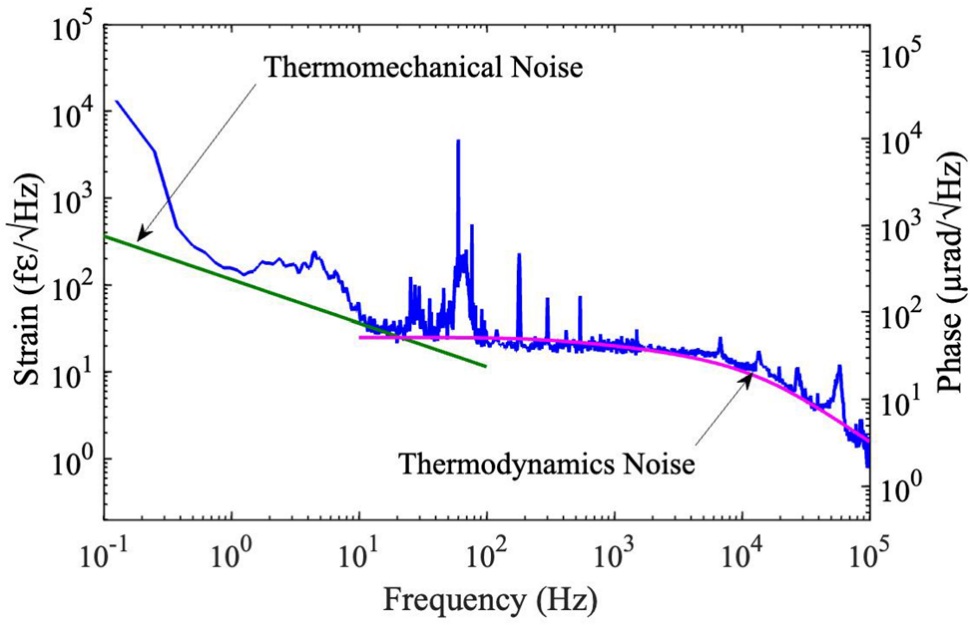
Noise-limited resolution of the MZ-FP interferometer (in strain and phase). Also shown are theoretical predictions of the noise floor due to fiber thermal noise, which indicates thermal-noise-limited operation at frequencies above 10 Hz.

Noise-limited resolution is a key parameter for an IFS. The noise floor of the MZ-FP interferometer has been captured with the DSA over a six-decade span of Fourier frequencies (0.1 - $10^5$ Hz). It is shown in Fig. 4 in terms of both strain and phase resolutions. The strain resolution is obtained by calibrating the measured noise spectrum (in unit of dBV/$\sqrt{Hz}$) using the linear strain response slope given in Fig. 3. The phase resolution is calculated from the strain resolution based on the relation $\delta \varphi = 2\pi (l/\lambda )\varepsilon$, where *l* is the effective arm length of the interferometer, $\lambda$ is wavelength, and $\varepsilon$ is strain. Note that *l* is related to the length of the FFPI $l_c$ through the relation $l=(2/\pi )\mathscr {F}l_c$, where $\mathscr {F}$ is the finesse of the FFPI^40^. The phase noise scale is also independently verified through a phase calibration process, which has been used in plotting Fig. 2b inset, and the result agrees with the calculated result very well. Based on Fig. 4, the noise spectrum features a gradual roll-off at high frequencies (> 1 kHz), a relatively flat region in the mid-frequency range (10 Hz–1 kHz), and a quick roll up at the low-frequency end (< 10 Hz), which qualitatively match the general behaviors of the fiber thermal noise^39^.

In order to make a quantitative comparison between the measured noise spectrum and the theoretical prediction of fiber thermal noise, both the thermodynamic noise and the thermomechanical noise are computed based on the established models^29^. The computation follows the strategy outlined by Duan for the MZ-FP configuration^39^ and uses parameters specific for SMF-28 fibers, including an effective refractive index of 1.468, a temperature coefficient for refractive index of $9.2\times 10^-{^6}$/K, a thermal expansion coefficient of $5.5\times 10^-{^7}$/K, a thermal conductivity of 1.37 W/(mK), a thermal diffusivity of $8.2\times 10^-{^7}$ m$^2$/s, boundary condition parameters of $3.846\times 10^5$/m and $3.848\times 10^4$/m, and a Young’s modulus of 68 GPa^36,42^. The resulted theoretical curves are also shown in Fig. 4. It is evident from Fig. 4 that the measured noise spectrum of our MZ-FP interferometer matches very well with the thermodynamic noise spectrum at Fourier frequencies above 10 Hz, indicating the attainment of thermal-noise-limited resolution in this frequency range. Below 10 Hz, however, the noise floor shows clear signs of low-frequency drift and remains above the predicted thermomechanical noise. Note that the noise peaks at 60 Hz, 180 Hz, 300 Hz and 540 Hz in the experimental trace are likely due to leaked power-line noise, while the noise bumps around 30 Hz and 5 Hz are believed to be caused by residual imbalance between the two FFPIs of mechanical and/or electronic origins. The high-frequency peak at 58 kHz is introduced by the PDH lockbox. It is worth mentioning here that a similar effort based on a *single* meter-long FFPI sensor has also been carried out but has failed to reach the thermal noise limit due to the dominance of laser noise^22,23^. This highlights the advantage of the MZ-FP hybrid scheme as it greatly mitigates the impact of the laser noise.

With the noise floor of the MZ-FP interferometer approaching the limit set by the fiber thermal noise, record-high strain resolutions have been achieved. Table 1 lists the measured strain resolutions at six decadal frequencies between 1 Hz and 100 kHz (top row) as well as the best results previously reported at these frequencies^23,37,38^. Note that some of the prior records are *estimated* based on graphical results because exact values of the strain resolution at these frequencies are not given in these reports. It is evident from Table 1 that the MZ-FP hybrid interferometer has achieved record-high strain resolutions across a broad frequency span (with the only exception at 1 kHz). In some instances, e.g., at 100 kHz, the improvement from previous record is nearly a factor of 10. These results demonstrate the superiority of the MZ-FP hybrid configuration as a scheme for ultrahigh-resolution fiber-optic sensing.

**Table 1:** Strain resolutions of the MZ-FP interferometer versus previous best results over six decades of frequencies.

Frequency (Hz)	1	10	$10^2$	$10^3$	$10^4$	$10^5$
Strain(f$\varepsilon$/$\sqrt{Hz})$	156	40	20	19	11	1
Prior Best Results (f$\varepsilon$/$\sqrt{Hz})$	$\sim$ 500^38^	$\sim 70$^38^	$\sim 20$^38^	14^38^	37^23^	$\sim 10$^37^

## Conclusion

In summary, we report the development of a new type of IFS, which is built upon a MZ-FP hybrid configuration. By using identical FFPIs as optical-path multipliers and with the help of a soil-based insulation system, the interferometer has demonstrated the capability of reaching extremely high resolutions limited only by the intrinsic thermal noise in optical fibers across a broad frequency range. The noise-limited strain resolutions are found to be 40 f$\varepsilon$/$\sqrt{(}Hz)$ at 10 Hz and 1 f$\varepsilon$/$\sqrt{(}Hz)$ at 100 kHz, which is by far the best strain resolution ever reported for an IFS. The unique hybrid scheme allows the MZ-FP interferometer to combine the benefits of both a double-path configuration and optical resonators, enabling thermal-noise-limited operation with only off-the-shelf components. With a proper scale-up, it is conceivable that *atto-strain* resolutions can be readily attained within the ultrasonic frequency range with such hybrid configurations. Thus, it is our hope that this work lays out a feasible path toward future atto-strain IFS.

## Data Availability

The authors declare that the data supporting the findings of this study are available within the article. All other relevant data are available from the corresponding author upon reasonable request.
